# Clinical Study on the Treatment of Female Stress Urinary Incontinence With Modified Buzhong Yiqi Decoction

**DOI:** 10.3389/fsurg.2022.882621

**Published:** 2022-04-13

**Authors:** Feifei Zhou, Chen Chen, Jiani Shi, Qianru Zhou, Lijun Jin, Xiaofang Ma

**Affiliations:** Department of Traditional Chinese Medicine, Center for Reproductive Medicine, Zhejiang Provincial People's Hospital (Affiliated People's Hospital, Hangzhou Medical College), Hangzhou, China

**Keywords:** Modified Buzhong Yiqi Decoction, female, stress urinary incontinence, efficacy, correlation

## Abstract

**Purpose:**

To study the clinical application value of Modified Buzhong Yiqi Decoction in the treatment of female stress urinary incontinence (SUI).

**Methods:**

A total of 103 female patients with SUI were included in this study, 13 were lost to follow-up, and the final number of studies was 90. General information about the patients, including age, years of menopause, body mass index (BMI), reproductive history, chronic respiratory disease, hypertension, and diabetes, were recorded. All the patients were treated with Modified Buzhong Yiqi Decoction alone for 4 weeks. The Patient Global Impression of Improvement (PGI-I), the International Consultation on Incontinence Questionnaire-Urinary Incontinence Short Form (ICI-QSF) and 72-h voiding diary were used to evaluate the patients' subjective symptoms and urinary incontinence degree before treatment, 1 month after treatment and 1 year after treatment, the efficacy and efficacy-related factors of Modified Buzhong Yiqi Decoction in the treatment of female SUI were analyzed.

**Results:**

One month after Modified Buzhong Yiqi Decoction treatment, compared with before treatment, the PGI-I questionnaire was very much better (68.89%), much better (8.89%), a little better (12.33%), no change (8.89%), the ICI-QSF score decreased (*P* < 0.05), and 72-h urine leakage frequency decreased (*P* < 0.05); One year after treatment compared with before treatment, the PGI-I questionnaire was very much better (40.00%), much better (17.78%), a little better (12.22%), no change (30.00%), the ICI-QSF score decreased (*P* < 0.05), and 72-h urine leakage frequency decreased (*P* < 0.05); and 1 year after treatment compared with 1 month after treatment, the ratio of very much better at 1 year after treatment was significantly decreased (*P* < 0.05), the score of the ICI-QSF was significantly increased (*P* < 0.05), and 72-h urine leakage frequency was significantly increased (*P* < 0.05). The correlation analysis showed that the efficacy at 1 month after treatment was negatively correlated with the severity of SUI and chronic respiratory diseases, but was not significantly correlated with age, menopause status, BMI, number of pregnancies, and number of births. The efficacy at 1 year after treatment was negatively correlated with the severity of SUI, chronic respiratory disease, age, and number of births and was positively correlated with BMI, but not significantly correlated with menopause status and number of pregnancies.

**Conclusion:**

Modified Buzhong Yiqi Decoction can effectively treat SUI in women. The efficacy is related to the severity of SUI and chronic abdominal hypertension, but the long-term efficacy decreases.

## Introduction

Stress urinary incontinence (SUI) is a disorder of voiding dysfunction due to anatomical defects in the supporting tissues of the urethra (1). Its clinical manifestations are involuntary urine flow when abdominal pressure increases such as coughing, sneezing, laughing, and exertion, but there is no enuresis in normal state, which affects the quality of life of more than 200 million people in the world (2). The disease is common in adult women and is the most common type of urinary incontinence. The prevalence of SUI in Chinese women has been reported to be as high as 18.9%, while the prevalence in older women over 60 years of age rises to about 30% (3). The incidence of SUI is showing a gradual increase with the advent of an aging population. The International Consultation on Incontinence (ICI) and the United Kingdom's National Institute for Health and Clinical Excellence (NICE) recommended nonsurgical treatment as the preferred treatment for SUI in women (3, 4).

At present, the study on the treatment of female SUI by traditional Chinese medicine (TCM) is limited to short-term clinical efficacy reports. So far, there is no clinical short-term and long-term efficacy observation and related drug pharmacological mechanism study. Chinese medicine believes that the operation of the spleen is mainly based on the rise and the rise is to maintain the normal position of the body's internal organs. “Su Wen Linglan Secret Canon” says: “The bladder is the official of the state capital, the body fluid is stored, and after the body fluid is vaporized by the yang of the lower coke, it can go out through the waterway.” The Kidney Qi controls stool and urination. If the Kidney Qi is insufficient and the Kidney Yang is deficient, the transformation of the Qi of the bladder is not smooth and symptoms such as clear and long urination last drops and urinary incontinence will be seen. Therefore, the clinical differentiation of SUI is not simply based on spleen deficiency, but mainly based on spleen and kidney deficiency and the principle of treatment is to invigorate the middle and ascend the sag and invigorate the kidney and solidify the astringency. In this study, the modified formula was prepared on the basis of Buzhong Yiqi Decoction and the Patient Global Impression of Improvement (PGI-I), the International Consultation on Incontinence Questionnaire-Urinary Incontinence Short Form (ICI-QSF), and 72-h urination diary were used. The short-term therapeutic effect of 1 month after treatment of Modified Buzhong Yiqi Decoction and the long-term therapeutic effect of 1 year after treatment were observed, to evaluate the feasibility and influencing factors of clinical application of this prescription in the treatment of female SUI. The report is as follows.

## Materials and Methods

### Study Object

This study was a prospective self-controlled study. A total of 103 female patients with SUI who were treated with Modified Buzhong Yiqi Decoction in the Pelvic Floor Disease Diagnosis and Treatment Center of the Obstetrics and Gynecology Hospital Affiliated to Zhejiang University School of Medicine from January 2012 to January 2015 were included.

### Western Medicine Diagnostic Criteria

It met the diagnostic criteria of the International Urogynecological Association (IUGA) for SUI in women (5). It is mainly manifested in the involuntary flow of urine from the urethral opening when the patient has a sudden increase in abdominal pressure. Urodynamic studies revealed abnormal results on uroflow rate, bladder manometry during filling, and stress leaky point pressure.

### Traditional Chinese Medicine Syndrome Differentiation

According to the theory of traditional Chinese medicine and related literatures, this study divided SUI into three syndrome types: Spleen-deficiency and Qi-trapping type, Kidney-Qi deficiency type, and Spleen-Kidney Yang deficiency type.

### Stress Urinary Incontinence Severity Rating

All the patients were graded according to the Ingelman–Sundberg scale (6): Mild: Urinary incontinence occurred when coughing and sneezing, no needed to use a urine pad. Moderate: Urinary incontinence occurred during daily activities such as running, jumping, and brisk walking and required the use of urine pads. Severe: Urinary incontinence occurs with light activity and changes in the supine position.

### Inclusion Criteria

It meets the diagnostic criteria for SUI. Be 18 years of age or older. Patients voluntarily accepted the treatment with Modified Buzhong Yiqi Decoction and subsequent clinical observation and signed the informed consent form. The patient was never referred for this. Urinary incontinence medication should be discontinued for more than 3 months.

### Exclusion Criteria

It includes residual urine > 30 ml and maximum urinary flow rate <20 ml/s. Other types of urinary incontinence, such as neurogenic bladder, psychogenic incontinence, and impulsive incontinence, are also present. Urethral sphincter atresia insufficiency, ectopic ureter, etc., requiring surgical treatment. Patients with urinary stones, infections, lower urinary tract obstruction, and renal disease. Patients with history of surgical treatment for urinary incontinence or history of pelvic floor surgery. Genital prolapse ≥ 2°. Combined with serious medical diseases such as cardiovascular and cerebrovascular, hematopoietic system, liver, and kidney. Reluctance to accept clinical observers. Those who did not adhere to the medication as prescribed and could not judge the curative effect. Those who could not meet the follow-up conditions.

### Medication Method

All the patients were treated with Modified Buzhong Yiqi Decoction orally. The main ingredients of Modified Buzhong Yiqi Decoction: 15 g of *Astragalus*, 15 g of fried *Atractylodes*, 6 g of tangerine peel, 6 g of *Cimicifuga*, 9 g of Bupleurum, 15 g of ginseng, 3 g of Licorice, 12 g of *Angelica*, 15 g of Alpiniae Oxyphyllae Fructus, 9 g of *Mantidis ootheca*, 15 g of Cortex Eucommiae, 6 g of *Lindera aggregata*, and 15 g of *Rosa laevigata* Michx. There were slight additions and subtractions through dialectics. Oral, 1 dose per day, warmly divided into two decoctions, each decoction was about 200 ml. Decoction in the morning and evening, 4 weeks as a course of treatment. The medicines were provided by the Chinese Medicine Pharmacy of the Obstetrics and Gynecology Hospital Affiliated to Zhejiang University School of Medicine, China, and each patient was treated for one course of treatment.

### Data Collection

In this study, a special person was responsible for recording the general conditions of the patients, including age, menopause, year of menopause, body mass index, reproductive history, whether there was a history of chronic increased abdominal pressure such as chronic respiratory disease, whether there was a history of common medical diseases in the elderly such as hypertension and diabetes mellitus; and follow-up was performed during and after taking the medicine for up to 1 year. Before treatment, 1 month after treatment and 1 year after treatment, the PGI-I was used to evaluate the patients' subjective symptoms and the ICI-QSF and 72-h urination diary were used to evaluate the degree of urinary incontinence. The ICI-QSF questionnaire included a total of 7 questions and each question had a score of 0 to 3. The higher the score, the greater the impact of urinary incontinence on the quality of life. The urination diary was used to continuously record urination for 72 h, including each urination time, urine volume, water drinking time, water consumption, accompanying symptoms, and urinary incontinence time, etc. In this study, a total of 103 female patients with SUI were treated with Modified Buzhong Yiqi Decoction, 13 patients with SUI were lost to follow-up, and 90 patients with SUI were finally studied.

### Data Analysis

Measurement data were described by mean ± SD, including age, height, weight, BMI, frequency of urinary leakage, and the ICI-QSF. The enumeration data were described by percentage, including age composition ratio, menopause or not and its years, reproductive history, chronic respiratory diseases, hypertension, and diabetes. Data analysis was performed using the statistical software SPSS version 11.0 (SPSS, Chicago, Illinois, USA). The ICI-QSF questionnaire score results and the number of urine leakage before and after treatment were analyzed by the *t*-test and the correlation between the treatment effect and various clinical characteristics was analyzed by bivariate correlation. A two-sided test with *P* < 0.05 was considered as statistically significant.

## Results

### Basic Information of Patients With Stress Urinary Incontinence

All the 103 patients with SUI completed 28-day treatment as required, of which 13 patients with SUI were lost to follow-up within 1 year after treatment, with a loss-to-follow-up rate of 12.62%. Lost telephone contact was the reason for the loss to follow-up. Data were analyzed using per-protocol (PP) analysis and those who were lost to follow-up were not included in the analysis. Among 90 patients who were followed-up to 1 year after treatment, age 50.04 ± 13.58 (years old), height 1.60 ± 0.05 (m), weight 60.25 ± 11.83 (kg), and BMI 23.46 ± 4.52 (kg/m^2^) were also included. The basic information of 90 patients with SUI was shown in Table 1.

**Table 1:** Basic information of patients with stress urinary incontinence (SUI) (*n* = 90).

**Items**	**Distributed**	**Number of people (ratio of the total)**
Age	<40 years old	19 (21.11%)
	40–50 years old	30 (33.33%)
	51–60 years old	22 (24.45%)
	>60 years old	19 (21.11%)
Menopause	1–5 years	17 (18.89%)
	6–10 years	7 (7.78%)
	>10 years	16 (17.78%)
Number of pregnancies	1 time	14 (15.56%)
	2 time	32 (35.56%)
	3 time	18 (20.00%)
	>3 time	26 (28.90%)
Number of births	1 time	49 (54.45%)
	2 time	30 (33.33%)
	3 time	7 (7.78%)
	>3 time	4 (4.44%)
Vaginal delivery		85 (94.44%)
Cesarean section		5 (5.56%)
Chronic abdominal hypertension disease	Chronic respiratory diseases	14 (15.56%)
Medical comorbidities	Hypertension	13 (14.44%)
	Diabetes	3 (3.33%)

### Comparison of the Patient Global Impression of Improvement at 1 Month and 1 Year After Treatment With Modified Buzhong Yiqi Decoction

**Figure 1:**
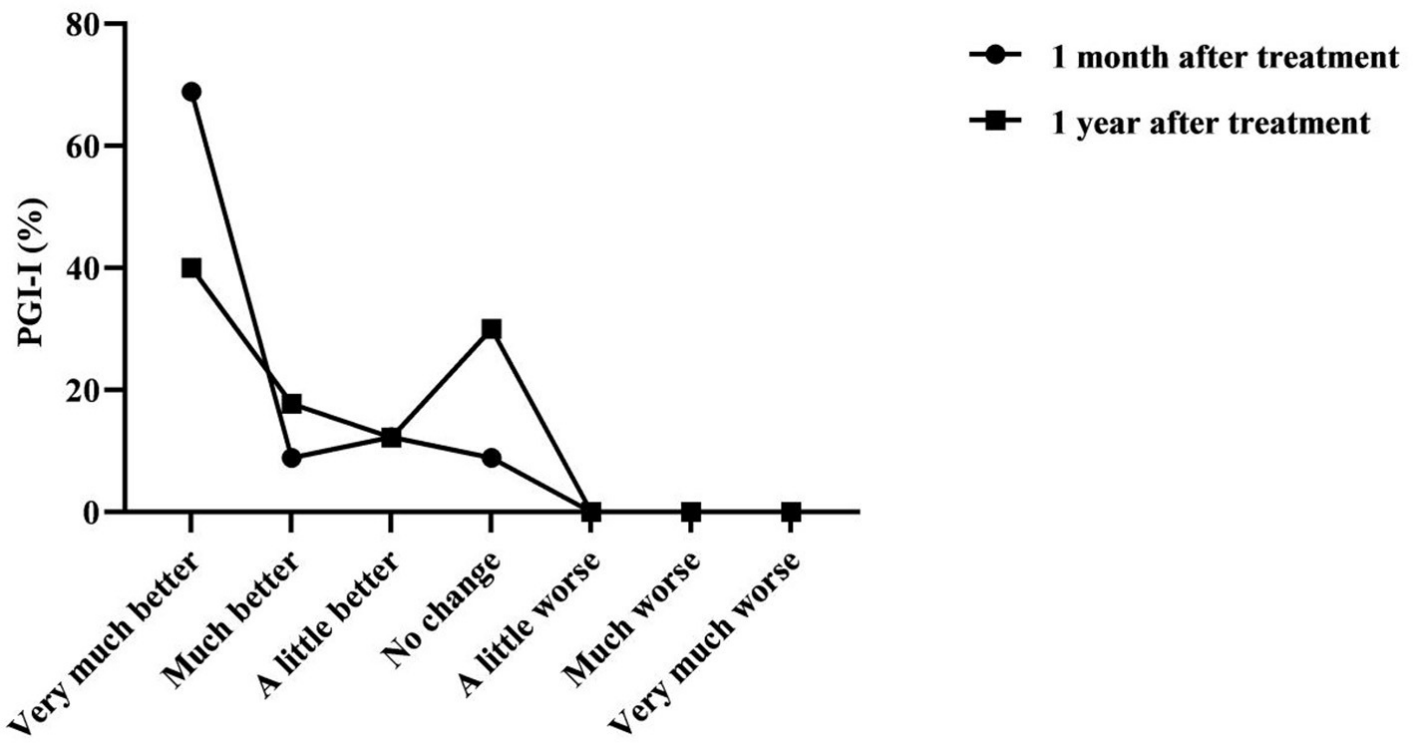
The Patient Global Impression of Improvement (PGI-I) at 1 month and 1 year after treatment with Modified Buzhong Yiqi Decoction (*n* = 90).

According to the PGI-I questionnaire, Table 2 and Figure 1 show that, compared with before treatment, the subjective symptoms of urinary incontinence were significantly improved after the treatment of Modified Buzhong Yiqi Decoction for 1 month and 1 year (*P* < 0.05); Compared to 1 month after treatment, there was a tendency for subjective symptoms of urinary incontinence to return 1 year after treatment: The self-reported no change and very much better 1 month after treatment were 8.89 and 68.89%, respectively, and the self-reported no change and very much better 1 year after treatment were 30.00 and 40.00%, respectively, and there was a statistical difference between the two (*P* < 0.05).

**Table 2:** The Patient Global Impression of Improvement (PGI-I) at 1 month and 1 year after treatment with Modified Buzhong Yiqi Decoction (*n* = 90).

**Choose a number that best described the condition of the patient's urethra after treatment compared to before the patient started treatment**.			
**Sequence**	**Description**	**1 month after treatment**	**1 year after treatment**
1	Very much better	62 (68.89)	36 (40.00)
2	Much better	8 (8.89)	16 (17.78)
3	A little better	12 (12.33)	11 (12.22)
4	No change	8 (8.89)	27 (30.00)
5	A little worse	0 (0.00)	0 (0.00)
6	Much worse	0 (0.00)	0 (0.00)
7	Very much worse	0 (0.00)	0 (0.00)

### Comparison of the International Consultation on Incontinence Questionnaire-Urinary Incontinence Short Form Score and 72-H Urine Leakage Frequency at 1 Month and 1 Year After Treatment With Modified Buzhong Yiqi Decoction

**Figure 2:**
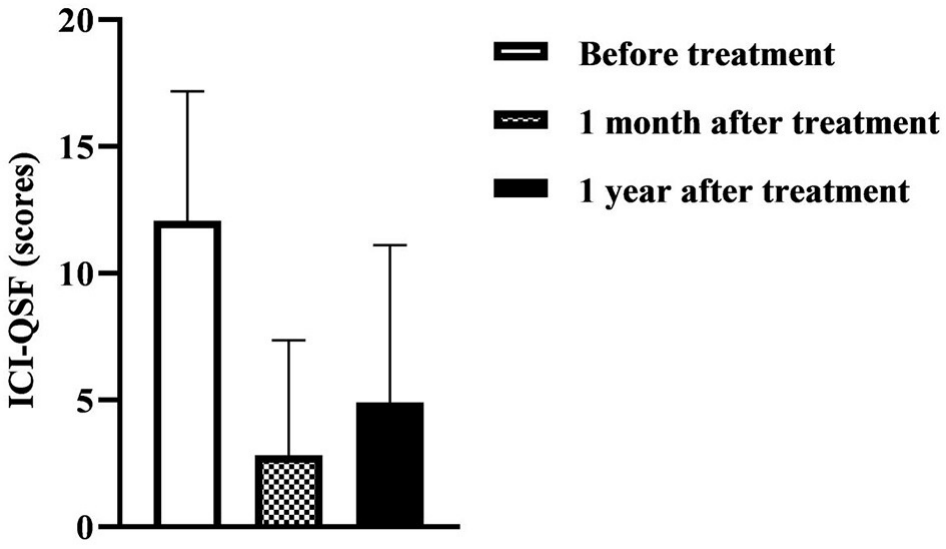
The International Consultation on Incontinence Questionnaire-Urinary Incontinence Short Form (ICI-QSF) score at 1 month and 1 year after treatment with Modified Buzhong Yiqi Decoction (*n* = 90).

**Figure 3:**
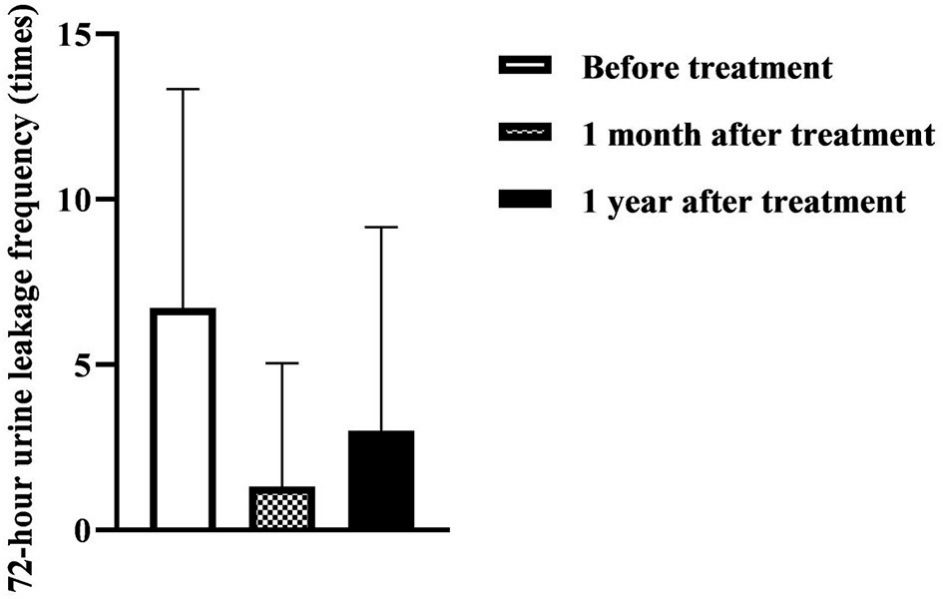
72-h urine leakage frequency at 1 month and 1 year after treatment with Modified Buzhong Yiqi Decoction (*n* = 90).

Table 3 and Figure 2 show that the ICI-QSF score before treatment with Modified Buzhong Yiqi Decoction was 12.08 ± 5.10, the ICI-QSF score at 1 month after treatment was 2.82 ± 4.53, and the ICI-QSF score at 1 year after treatment was 4.90 ± 6.21. The latter two were significantly lower than those before treatment (*P* < 0.05), but the ICI-QSF score at 1 year after treatment was significantly higher than that at 1 month after treatment (*P* < 0.05). Table 3 and Figure 3 show that 72-h urine leakage frequency before treatment with Modified Buzhong Yiqi Decoction was 6.72 ± 6.61, 72-h urine leakage frequency at 1 month after treatment was 1.32 ± 3.72, and 72-h urine leakage frequency in 1 year after treatment was 3.00 ± 6.16, the latter two were also significantly lower than those before treatment (*P* < 0.05), and 72-h urine leakage frequency at 1 year after treatment was significantly higher than that at 1 month after treatment (*P* < 0.05).

**Table 3:** The International Consultation on Incontinence Questionnaire-Urinary Incontinence Short Form (ICI-QSF) score and 72-h urine leakage frequency at 1 month and 1 year after treatment with Modified Buzhong Yiqi Decoction (*n* = 90).

	**Before treatment**	**1 month after treatment**	**1 year after treatment**
ICI-QSF (scores)	12.08 ± 5.10	2.82 ± 4.53*	4.90 ± 6.21^*#^
Frequency of urine leakage (times)	6.72 ± 6.61	1.32 ± 3.72*	3.00 ± 6.16^*#^

*was a significant difference compared with before treatment, P < 0.05; # was a significant difference compared with 1 month after treatment, P < 0.05.

### Analysis of the Related Factors of Curative Effect of Modified Buzhong Yiqi Decoction in the Treatment of Female Stress Urinary Incontinence

Using the PGI-I to evaluate the treatment effect, after statistical analysis of correlation, Table 4 shows that, 1 month after treatment, the curative effect was negatively correlated with SUI severity and chronic respiratory disease (*P* < 0.05), but had no significant correlation with age, menopause situation, BMI, number of pregnancies, and number of births (*P* > 0.05). 1 year after treatment, the curative effect was negatively correlated with SUI severity, chronic respiratory disease, age, and number of births (*P* < 0.05) and was positively correlated with BMI (*P* < 0.05), but had no significant correlation with menopause situation and number of pregnancies (*P* > 0.05).

**Table 4:** Analysis of related factors of curative effect of Modified Buzhong Yiqi Decoction after 1 month and 1 year of treatment.

**Factor**	**1 month after treatment**		**1 year after treatment**	
	**Spearman correlation coefficient**	***P* value**	**Spearman correlation coefficient**	***P* value**
Age	−0.136	0.314	−0.446	<0.001
Menopause	0.197	0.141	0.222	0.1
Year of menopause	−0.157	0.243	−0.188	0.165
BMI	−0.007	0.96	0.568	<0.001
Number of pregnancies	−0.006	0.962	−0.086	0.530
Number of births	−0.02	0.884	−0.215	0.023
SUI severity	−0.51	<0.001	−0.478	<0.001
Chronic respiratory diseases	−0.392	0.003	−0.38	0.004

### Safety of Modified Buzhong Yiqi Decoction in the Treatment of Female Stress Urinary Incontinence

There were no adverse reactions during and 1 year after taking the drug, including 19 elderly patients > 60 years old and 16 patients with hypertension or diabetes and no aggravation of primary underlying diseases such as hypertension and diabetes was found.

## Discussion

Urinary incontinence can lead to inconvenience in movement, inability to perform housework, affect normal social and sexual life, etc., resulting in the isolation of patients from society and family and a series of depression or psychological disorders (7). According to a community survey of women over the age of 40 years in the United Kingdom, the prevalence of urinary incontinence in the region was 34%, but only 25% of patients visited a doctor. A survey in Germany showed that 43% of patients concealed a history of urinary incontinence and even 25% of patients had a history of more than 5 years. Thus, urinary incontinence is not only a health problem, but also a social problem. SUI is the most common type of urinary incontinence, accounting for about 50% of urinary incontinence and its economic burden on families and society cannot be ignored (8, 9). At present, the treatment methods of SUI mainly include drug therapy, surgery, physical therapy, and so on. Among them, surgical treatment has many adverse reactions and although drugs and physical therapy have few adverse reactions, the cure rate is still unsatisfactory (10).There is still a need to find better ways to treat SUI.

The understanding of urinary incontinence in Chinese medicine has a long history; water and fluid are controlled in the spleen; if the Spleen Qi does not rise, urinary incontinence is seen; however, its origin is in the kidney. If the Kidney Qi is insufficient and the Kidney Yang is deficient, urinary incontinence can also be seen. Therefore, to identify its etiology and pathogenesis, it must be that the Spleen Qi does not rise, the Qi transformation has no power, the Kidney Qi is insufficient, the bladder is out of restraint, and the opening and closing are abnormal. Urinary incontinence mostly occurs in elderly patients. Traditional Chinese medicine believes that when a person reaches the age of 49 years, the Ren pulse is deficient, the Taichong pulse is less weak, and the Kidney Qi is weak. Simple application of Buzhong Yiqi Decoction cannot improve the symptoms of urinary incontinence caused by kidney deficiency. Therefore, in this study, on the basis of Buzhong Yiqi Decoction, the medicine for nourishing Kidney Qi is added, the treatment is to nourish the middle and ascend the sag, invigorate the kidney, solidify astringency, and after a 1-year follow-up study, in order to evaluate the clinical application value of Modified Buzhong Yiqi Decoction in the treatment of female SUI.

Compared with before treatment, 1 month after treatment with Modified Buzhong Yiqi Decoction, the subjective symptoms of the patients were significantly different (*P* < 0.05), the ICI-QSF score was decreased (*P* < 0.05), and 72-h urine leakage frequency was decreased (*P* < 0.05). It is indicated that by invigorating the middle and ascending the sag and invigorating the kidney and solidifying the astringency, the Modified Buzhong Yiqi decoction can effectively improve the symptoms of female SUI. One year after treatment, compared with before treatment, there were still significant differences in patients; subjective symptoms (*P* < 0.05), the ICI-QSF score still decreased (*P* < 0.05), and 72-h urine leakage frequency still decreased (*P* < 0.05). It shows that Modified Buzhong Yiqi Decoction is still effective for female SUI 1 year after treatment compared with before treatment. However, 1 year after treatment compared with 1 month after treatment, the rate of very much better at 1 year after treatment was significantly lower (*P* < 0.05), the ICI-QSF score was significantly higher (*P* < 0.05), and 72-h urine leakage frequency was significantly increased (*P* < 0.05). It shows that the improvement of SUI symptoms and quality of life with Modified Buzhong Yiqi Decoction cannot be permanent and the efficacy decreases with time. In addition, we found that the short-term (1 month) and long-term (1 year) efficacy of Modified Buzhong Yiqi Decoction for severe SUI were relatively poor. Based on this, we speculate that Modified Buzhong Yiqi Decoction is suitable for mild-to-moderate female SUI and patients with recurrent disease need to increase the course of treatment to consolidate the curative effect. Among the cases, 2 patients had SUI symptoms after repregnancy and the statistics were judged as no change, but they were still cured after using Modified Buzhong Yiqi Decoction again.

In the formula of Modified Buzhong Yiqi Decoction, *Astragalus* enters the spleen and lung meridians, can invigorate the middle and benefit qi, raise yang and ascend sag, and can solidify the surface and stop sweating, so it is reused as the king (11). Ginseng is great for nourishing vitality, fried *Atractylodes* nourishes Qi and spleen, Licorice nourishes the middle and invigorating Qi, and helps *Astragalus* to nourish Qi and strengthen the spleen, all of which are ministerial medicines (12, 13) and the patient's qi deficiency for a long time often damages the blood. Therefore, it is compatible with *Angelica* to nourish blood and nutrition; tangerine peel can adjust Qi and regulate Qi and stomach, so that various medicines can be replenished without stagnation; *Cimicifuga* and *Bupleurum* can help to raise yang and ascend sag, which can help the monarch medicine to ascend the Qi in depression; Alpiniae Oxyphyllae Fructus, *Mantidis ootheca*, and *Lindera aggregata* warm the spleen and kidney; Cortex Eucommiae nourishes the liver and kidney; *Rosa laevigata* Michx shrinks urine and solidifies sperm. The above flavors are used as adjuvants and are basically suitable for various syndromes of SUI (14–16). Licorice reconciles various medicines and is also used as messenger medicine. This formula combines tonification with Qi circulation, tonifying but not accumulating stagnation, and tonification with elevation, raising yang and lifting traps, which is especially suitable for Spleen and Stomach Qi deficiency and lower detachment (17).

This study found that chronic respiratory diseases were negatively correlated with the efficacy of Modified Buzhong Yiqi Decoction at 1 month and 1 year after treatment. It shows that if the patient is complicated with chronic respiratory system diseases, the short-term and long-term efficacy will be poor, suggesting that active treatment of chronic abdominal pressure diseases such as chronic abdominal hypertension disease should be carried out while traditional Chinese medicine is used to treat female SUI. This study also found that with the increase of age and the number of births, the long-term efficacy of Modified Buzhong Yiqi Decoction in the treatment of SUI was worse. It is well known that urethral support tissue damage and degeneration caused by pregnancy and childbirth and menopause and aging are important causes of SUI in women. Prolificity can cause repetitive, stale damage to the supporting tissues of the urethra. Aging can cause irreversible degenerative changes in the supporting tissues of the urethra. Therefore, the efficacy of drug intervention in such patients is relatively reduced. However, we did not find a correlation between menopause and the efficacy of Modified Buzhong Yiqi Decoction in the treatment of SUI, which may be related to the fact that the average age of the population included in this study was 50 years old and it was mainly perimenopausal and postmenopausal women. In addition, we found that BMI was positively correlated with the efficacy of Modified Buzhong Yiqi Decoction in the treatment of SUI. BMI is a high risk factor for SUI and people with high BMI are prone to spleen deficiency (18–20). From this, we speculate that Modified Buzhong Yiqi Decoction has the effect of invigorating the spleen and the spleen is healthy and normal, the water transport and transformation will be normal, so it is suitable for patients with SUI with obesity.

Stress urinary incontinence is more common in middle-aged and elderly women and elderly patients often have medical diseases such as hypertension and diabetes and their drug tolerance decreases (21). For example, Western medicine α1-adrenoceptor agonists tend to constrict vascular smooth muscle and affect hypertensive patients (22). Tricyclic antidepressants have uncertain efficacy and many side effects, including nausea and vomiting (40% or more of patients), dry mouth, constipation, dizziness, insomnia, somnolence, and fatigue (23). Therefore, there is no recognized effective and safe anti-SUI western medicine. In this study, there were 19 elderly patients over 60 years old and 16 patients with hypertension or diabetes. There were no adverse reactions during the medication and during the follow-up and no exacerbation of hypertension and/or diabetes was found. It shows that the safety of Modified Buzhong Yiqi Decoction is good and it is suitable for clinical promotion.

## Conclusion

Modified Buzhong Yiqi Decoction can effectively treat SUI in women. The efficacy is related to the severity of SUI and chronic abdominal hypertension, but the long-term efficacy decreases.

## Data Availability Statement

The original contributions presented in the study are included in the article/supplementary material, further inquiries can be directed to the corresponding author.

## Ethics Statement

The studies involving human participants were reviewed and approved by the Ethics Review Committee of the Obstetrics and Gynecology Hospital Affiliated to Zhejiang University School of Medicine. The patients/participants provided their written informed consent to participate in this study.

## Author Contributions

CC and JS are the mainly responsible for the writing of the article. QZ is mainly responsible for research design. FZ is mainly responsible for data analysis. LJ and XM are responsible for the guidance of the entire research. All authors contributed to the article and approved the submitted version.

## Funding

This study was supported by the Zhejiang Province TCM Key Project Research (2018ZZ017) and the Zhejiang Provincial Department of Education (Y202146113).

## Conflict of Interest

The authors declare that the research was conducted in the absence of any commercial or financial relationships that could be construed as a potential conflict of interest.

## Publisher's Note

All claims expressed in this article are solely those of the authors and do not necessarily represent those of their affiliated organizations, or those of the publisher, the editors and the reviewers. Any product that may be evaluated in this article, or claim that may be made by its manufacturer, is not guaranteed or endorsed by the publisher.
